# Latent Prototype-Based Clustering: A Novel Exploratory Electroencephalography Analysis Approach

**DOI:** 10.3390/s24154920

**Published:** 2024-07-29

**Authors:** Sun Zhou, Pengyi Zhang, Huazhen Chen

**Affiliations:** 1 Department of Automation, Xiamen University, Xiamen 361102, China; zhangpengyi@stu.xmu.edu.cn; 2 School of Sociology and Anthropology, Xiamen University, Xiamen 361005, China; chenhuazhen@stu.xmu.edu.cn

**Keywords:** EEG, GAN, clustering, GMM

## Abstract

Electroencephalography (EEG)-based applications in brain–computer interfaces (BCIs), neurological disease diagnosis, rehabilitation, etc., rely on supervised approaches such as classification that requires given labels. However, with the ever-increasing amount of EEG data, incomplete or incorrectly labeled or unlabeled EEG data are increasing. It likely degrades the performance of supervised approaches. In this work, we put forward a novel unsupervised exploratory EEG analysis solution by clustering based on low-dimensional prototypes in latent space that are associated with the respective clusters. Having the prototype as a baseline of each cluster, a compositive similarity is defined to act as the critic function in clustering, which incorporates similarities on three levels. The approach is implemented with a Generative Adversarial Network (GAN), termed W-SLOGAN, by extending the Stein Latent Optimization for GANs (SLOGAN). The Gaussian Mixture Model (GMM) is utilized as the latent distribution to adapt to the diversity of EEG signal patterns. The W-SLOGAN ensures that images generated from each Gaussian component belong to the associated cluster. The adaptively learned Gaussian mixing coefficients make the model remain effective in dealing with an imbalanced dataset. By applying the proposed approach to two public EEG or intracranial EEG (iEEG) epilepsy datasets, our experiments demonstrate that the clustering results are close to the classification of the data. Moreover, we present several findings that were discovered by intra-class clustering and cross-analysis of clustering and classification. They show that the approach is attractive in practice in the diagnosis of the epileptic subtype, multiple labelling of EEG data, etc.

## 1. Introduction

Electroencephalography (EEG) is a well-established non-invasive tool to record brain electrophysiological activity. Compared to other neuroimaging techniques that provide information about the anatomical structure (e.g., MRI, CT, and fMRI), EEG offers ultra-high time resolution, which is critical in understanding brain function. As the mainstream means for examining brain electrical activities, EEG techniques have wide applications in cognitive neuroscience, emotion recognition [1], motor imagery [2], and the diagnosis of diseases such as autism, schizophrenia, and epilepsy [3]. However, these applications are mostly focused on supervised tasks that require a priori knowledge such as EEG labels that define the class they belong to. However, not all EEG labels associated with specific patterns of brain activity can be completely or correctly obtained from subjects across different recording sessions. This is especially so for patients with complex situations, such as those suffering from stroke [4], Alzheimer’s disease (AD) [5], amyotrophic lateral sclerosis (ALS) [6], or epileptic seizures [7], etc. Therefore, the increasing amount of incomplete or incorrectly labeled or unlabeled EEG data likely degrades the efficacy of supervised techniques, e.g., classification that is crucial to brain–computer interface (BCI)-based applications and disease diagnosis. Moreover, in fact, the EEG signals often have multiple attributes, consequently needing to be annotated with multiple labels. Some EEG data may have subclasses that deserve more attentions in clinical applications, like epileptic subtype diagnosis. In practice, either multiple labelling or subclass labelling is almost infeasible due to the multiple labor and time costs of the labelling.

Dai et al. suggested a semi-supervised EEG clustering method that makes good use of limited a priori knowledge [8]. In our work, we focus on unsupervised clustering. Clustering aims to organize the data elements of a dataset into distinct clusters according to the resemblance of the intrinsic patterns of the data. Data elements of the same cluster are characterized by a similarity higher than those of other clusters [9]. Clustering is probably the most important and fundamental means of exploratory data analysis for finding intrinsic hidden information and patterns (if any) without requirement for a priori knowledge, such as to detect unknown kinds of abnormal states from brain imaging data.

The most widely applied clustering techniques, such as K-means, rely on distance to assign a cluster, which determine the cluster members to centers based on their minimum distances and find the most appropriate cluster centers by the optimization of an objective function based on distance [10]. Distance-based algorithms are the most commonly used benefiting from this simple principle.

To adapt to the diversity of data, distribution-based approaches such as the Gaussian Mixture Model (GMM) have drawn more attentions. They employ predefined probability distribution functions to reproduce data elements [11]. If the predefined distribution cannot be adaptively adjusted, the clustering efficacy relies on the capability of the trial probability in representing the data. Based on the Density Peaks Clustering (DPC) algorithm [12], Gao et al. formed an adaptive density peaks clustering (ADPC) solution towards exploratory EEG analysis [13].

Generative Adversarial Networks (GANs) have obtained remarkable success in many unsupervised learning tasks [14]. In recent times, in order to provide a better fit to the target data distribution when the image dataset includes many different classes, some variants of the basic GAN model, including Gaussian Mixture GAN (GM-GAN), dynamic GM-GAN [15], and Deli-GAN [16], have been proposed where the probability distribution over the latent space is a mixture of Gaussians. These models tend to map latent vectors sampled from different Gaussians in the latent space to samples of different classes in the image data space. This phenomenon implies that it may be exploited for the task of unsupervised clustering. However, these GANs do not provide inverse mapping from the data space ***X*** to the latent space ***Z***. Therefore, given a query data point, we cannot know which latent variable it is generated from, or to say, we cannot obtain its latent space representation.

Some GAN techniques make use of an encoder that has the potential to provide another form of back-projection, such as InfoGAN [17], Variational Auto-Encoder GAN (VAE-GAN) [18], and Stein Latent Optimization for GANs (SLOGAN) [19]. Usually, they are not specifically designed for clustering.

The main contributions of this work are as follows.

A novel unsupervised approach is put forward for exploratory EEG analysis. The basic idea is to form a kind of GAN to learn the Gaussian mixture distribution in latent space wherefrom the prototype or center associated with each cluster can be abstracted. Then, based on the latent prototypes, according to a well-defined similarity metric, the query EEG data will be assigned to a cluster.

By applying the proposed approach to two public EEG or intracranial EEG (iEEG) epilepsy datasets, our experiments demonstrate that the clustering results are close to the classification of the data. Moreover, several findings show that the approach is attractive in practice in the diagnosis of epileptic subtypes and multiple labelling of EEG data, etc.

## 2. Materials

In this work, two publicly available EEG epilepsy datasets were used in the experiments, the benchmark Bonn dataset and the HUP iEEG dataset.

### 2.1. Bonn Dataset

The Bonn dataset [20], collected by the University of Bonn, contains EEG and iEEG signals from healthy volunteers and epileptics. The muscle activity and eye movement artifacts were already removed from the collected data on the basis of visual inspection [21]. The complete database consists of by five sets denoted as A–E. Sets A and B contain scalp EEG signals collected from healthy volunteers with their eyes open (A) and closed (B). Set C contains iEEG recordings that were recorded from the hippocampal formation of opposite hemispheric regions during inter-ictal periods [22]. Set D comprises the iEEG signals collected from within the epileptic zone of the brain of patients during seizure-free intervals. Set E contains data collected from within the epileptogenic zone of patients during the ictal period. Detailed descriptions of the dataset are shown in Table 1. Each set contains 100 single-channel EEG or iEEG segments with a sampling rate of 173.61 Hz and a duration of 23.6 s.

### 2.2. HUP IEEG Epilepsy Dataset

**Figure 1:**
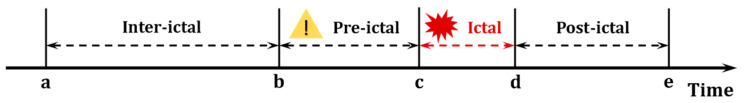
Different periods of electroencephalography (EEG) signals of an epileptic. a–e denote different time points.

The HUP dataset [23], collected by the Hospital of the University of Pennsylvania, contains intracranial EEG (iEEG) signals from 58 patients diagnosed as drug-resistant epilepsy. Each of the 58 subjects underwent iEEG with subdural grid, strip, and depth electrodes (electrocorticography (ECoG)) or purely stereotactically placed depth electrodes (sEEGs). Since each patient’s epilepsy type may not be the same, a patient-specific study is necessary [24]. We chose the ECoG signals of three de-identified patients, HUP65, HUP88, and HUP89, to be used in the experiments. Details of the dataset are provided in Table 2. The data for each patients include three ictal and two inter-ictal segments, stored in EDF format. Each ictal segment includes recordings from two minutes before the seizure onsets, which were viewed as the pre-ictal period in this study. Therefore, data of each patient can be categorized into three periods: pre-ictal, ictal, and inter-ictal. Figure 1 illustrates the different periods in EEG data collected from epileptics.

## 3. Methods

Given a test data, the probability for each cluster can be calculated using a given critic function, which enables us to assign a cluster for the data. We propose a critic function based on the test data’s low-dimensional prototype in the latent space, termed latent prototype. Each prototype is responsible for a certain attribute of the data, namely a cluster. Having the prototype as a baseline of each cluster, we are able to define a compositive critic metric that incorporates the similarities between the test data and the prototype of a given cluster on three levels, which are the latent representation level, image level, and deep feature map level.

### 3.1. Schematic of Latent Prototype-Based Clustering

**Figure 2:**
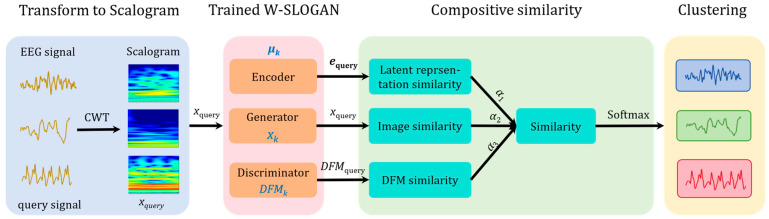
Schematic of EEG clustering solution based on latent prototypes. CWT, continuous wavelet transform. DFM, deep feature map. ***e***_query_, latent space representation of the query signal. $\mu_{k}$, latent prototype of the *k*th cluster. $x_{query}$, scalogram of the query signal. $x_{k}$, baseline scalogram of the *k*th cluster. ${DFM}_{query}$_,_ deep feature map of the query signal. ${DFM}_{k}$, baseline deep feature map of the *k*th cluster. $\alpha_{1}$, $\alpha_{2}$, and $\alpha_{3}$ are weights.

According to the above consideration, we put forward an unsupervised EEG clustering approach based on latent prototypes. Its schematic is briefly illustrated in Figure 2. First, train a W-SLOGAN from the EEG dataset to learn a generator, a discriminator, an encoder as well as the latent prototype $\mu_{k}$ that are responsible for each cluster. Given a query signal, transform it with continuous wavelet transform to a scalogram $x_{query}$. Wavelet transform is an effective technique to analyze the local characteristics of non-stationary signals, offering both time domain resolution and frequency domain resolution. Then, utilizing the trained W-SLOGAN, calculate three levels of similarities separately between (i) the latent space representation of the query signal and the latent prototype of each cluster, (ii) scalogram of the query signal and the baseline scalogram of each cluster, and (iii) the deep feature map (DFM) of the query signal and the baseline deep feature map of each cluster. Obtain the compositive similarity between the query signal and the prototype of each cluster by incorporating the above three levels of similarities. Finally, convert the compositive similarity to a probability with the SoftMax function, which enables us to assign a cluster for the query signal.

### 3.2. Gaussian Mixture Distribution in Latent Space

**Figure 3:**
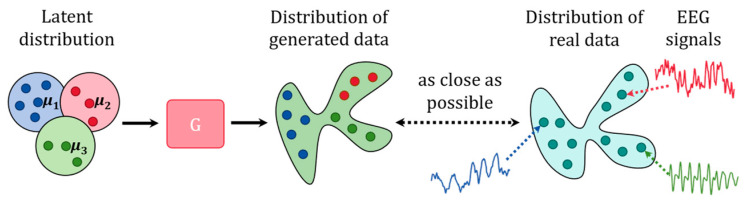
Latent distribution defined as Gaussian mixture distribution and distribution of generated data and that of real data. Suppose there are three clusters in the dataset. $\mu_{1}$, $\mu_{2}$, and $\mu_{3}$ can be regarded as the latent prototypes of the three clusters.

GANs usually uses a unimodal distribution as the prior distribution for *Z*, such as the multivariate uniform distribution (i.e., $U{\left[-1,1\right]}^{d_{z}}$) and the multivariate normal distribution (i.e., $N(0,I_{d_{z}\times d_{z}})$) [15]. To better adapt to diversity of the real data, the W-SLOGAN adopts the multimodal Gaussian mixture distribution as the prior to sample from latent space, as shown in Figure 3. 

The Gaussian mixture distribution is defined as follows:

(1) $$q(z)=\sum_{k=1}^{N}p(k)q(z|k)$$

where *N* is the number of Gaussian components and can be predefined by the number of data clusters, p(k) is mixing coefficient, and q(z|k) denotes the probability distribution of the *k*th Gaussian component, formulated as $q\left(z|k\right)=N(z;\mu_{k},\Sigma_{k})$, where $\mu_{k}$ and $\Sigma_{k}$ denote the mean vector and covariance matrix of the *k*th Gaussian component, respectively.

### 3.3. W-SLOGAN

We decided to form a kind of GAN to obtain the latent prototype of each cluster that is needed in the calculation of compositive similarity. GAN is a generative model that learns a generator (*G*) capable of generating samples from the data distribution ($p_{data}$), by converting latent vectors from a lower-dimension data space (*Z*) to samples in a higher-dimension data space (*X*). Specifically, we need a kind of GAN such that: (i) The latent space distribution of the GAN should be defined as a Gaussian mixture distribution to model the diversity of the data. (ii) It should be able to learn the latent prototype that is responsible for each cluster from the data distribution. (iii) It is able to back-project the query image to the latent space; therefore, an encoder (*E*) is needed. (iv) Well-defined objective functions are needed for training *G*, *D*, and *E* and the latent distribution. Based on the above consideration, we put forward a GAN, termed W-SLOGAN, which takes advantage of both the Wasserstein GAN with Gradient Penalty (WGAN-GP) and the Stein Latent Optimization for GANs (SLOGAN), especially the latter. The W-SLOGAN adopts a discriminator objective function and adversarial loss function proposed in WGAN-GP, with which the training can be more stable, and the generated images can be of better quality. Also, it utilizes an encoder as well as an Unsupervised Conditional Contrastive loss (U2C loss), ensuring that the encoded vector of the generated image is similar to its assigned low-dimensional prototype in the latent space.

In the following, the network architecture, objective functions, and optimization algorithm of the W-SLOGAN will be described.

#### 3.3.1. Network Architecture

**Figure 4:**
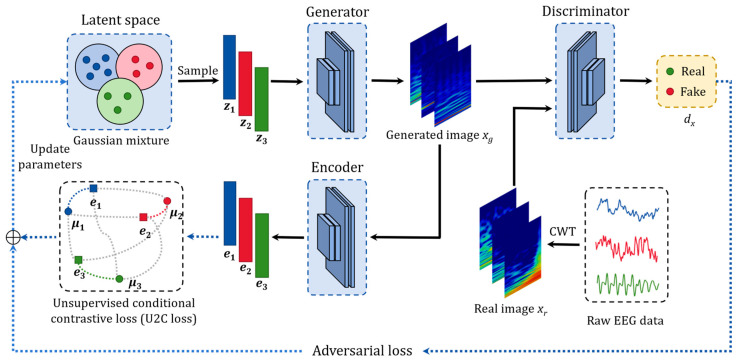
Network architecture of W-SLOGAN. The latent distribution is defined as Gaussian mixture distribution. Assume the number of Gaussian components is 3. $z_{1}$, $z_{2}$, and $z_{3}$ denote the latent vectors sampled from latent space. $e_{1}$, $e_{2}$, and $e_{3}$ denote the encoded vectors of the scalograms calculated by the encoder. $\mu_{1}$, $\mu_{2},$ and $\mu_{3}$ denote the mean vectors of the three Gaussian components, corresponding to the latent prototypes of the three clusters. $d_{x}$ denotes the output of the discriminator.

Figure 4 shows the network architecture of W-SLOGAN, which consists of a generator (*G*), a discriminator (*D*), and an encoder (*E*). *G* is responsible for mapping the latent space (*Z*) defined by Gaussian mixture distribution to the real image domain (*X*) ($G\left(z\right):Z\to X$). In this mapping, the mean vector $\mu_{k}$ of each Gaussian component in the latent space can be viewed as a prototype of samples with certain salient attribute, and the average representation of that attribute in the latent space. *D* receives the generated images ($x_{g}$) and real images ($x_{r}$) to train its capability to discriminate between real and fake, providing the driving force for the training of the generator. *E* maps the image onto a space of the same dimension as the latent space. In order to stabilize the process of adversarial learning and improve the learning of images, W-SLOGAN adopts the convolutional layer structure that was introduced in Deep Convolutional GAN (DCGAN) [25]. Table 3 provides the implementation details of the W-SLOGAN model.

#### 3.3.2. Objective Functions

W-SLOGAN is trained to learn the parameters of the generator *G*, discriminator *D*, and encoder *E* from the data, as well as the parameters ($\mu_{k}$, $\Sigma_{k}$, and p(k)) of the Gaussian mixture distribution of the latent space. It is necessary to define well the objective functions for the training. We chose a discriminator objective function and an adversarial loss function that were the same as those of WGAN-GP. The unsupervised conditional contrastive loss (U2C loss) proposed by Hwang et al. [19] for SLOGAN was also employed in the training. In the training of W-SLOGAN, *D* and (*G*, *E*, $\mu_{k}$, $\Sigma_{k}$, and p(k)) were updated alternately. *D* was trained with the discriminator objective function, and *G*, *E*, $\mu_{k}$, $\Sigma_{k}$, and p(k) were trained with a total objective function that comprises the adversarial loss and the U2C loss.

Discriminator objective function. The discriminator objective function is defined as that of WGAN-GP, which helps to stabilize the training process and provide the driving force for the training of generator [26,27,28]. It is defined as

(2) $$L_{D}={-E}_{x\in P_{data}}\left[D\left(x\right)\right]+E_{z\in q\left(z\right)}\left[D\left(G\left(z\right)\right)\right]+\lambda_{1}E_{\tilde{x}\in P_{penalty}}[{\left({\left\|\nabla_{\tilde{x}}D\left(\tilde{x}\right)\right\|}_{2}-1\right)}^{2}]$$

where $\lambda_{1}$ denotes the gradient penalty coefficient, and $\tilde{x}$ is sampled from the line between the real training data distribution and the generated data distribution. Such a design can make the model converge faster.

Adversarial loss function. The purpose of minimizing adversarial loss is to make the samples generated by the generator as realistic as possible, so that the discriminator cannot accurately distinguish between the generated samples and the fake ones. The adversarial loss function is defined as that of WGAN-GP, as formulated in (3).

(3) $$l_{adv}\left(z^{i}\right)=-D(G(z^{i})$$

Total objective function. W-SLOGAN learns the parameters of the generator (*G*), encoder (*E*), and the Gaussian mixture distribution of the latent space by minimizing the total objective function, including unsupervised conditional contrastive loss (U2C loss) and adversarial loss. The total objective function is defined as follows:

(4) $$L_{total}=\frac{1}{B}\sum_{i=1}^{B}\left(l_{adv}\left(z^{i}\right)+\lambda_{2}l_{U2C}\left(z^{i}\right)\right)$$

where $\lambda_{2}$ denotes the weight coefficient of the U2C loss.

U2C loss. With U2C loss, the training allows each salient attribute to cluster in the latent space, and each component of the learned latent distribution is responsible for a certain attribute of the data. Given a batch of latent vectors ${\left\{z^{i}\right\}}_{i=1}^{B}\;\sim \;q(z)$ (where *B* is the batch size), we can find the corresponding Gaussian component $K^{i}$ (the mean vector is $\mu_{K}^{i}$) to which $z^{i}$ is most likely belong by the use of (5).

(5) $$K^{i}={argmax}_{k}q\left(k|z^{i}\right)={argmax}_{k}\frac{q(k,z^{i})}{q(z^{i})}={argmax}_{k}\frac{q(z^{i}|k)p(k)}{q(z^{i})}$$

The generator receives the latent vector $z^{i}$ and generates the corresponding sample $x_{g}^{i}=G(z^{i})$. Then, the generated sample $x_{g}^{i}$ is mapped by the encoder to an encoded vector $x_{g}^{i}=G(z^{i})$. The cosine similarity between $e^{i}$ and $\mu_{K}^{j}$ can be calculated using $cos\theta_{ij}=e^{i}\cdot \mu_{K}^{j}/\left\|e^{i}\right\|\left\|\mu_{K}^{j}\right\|$. The U2C loss is defined as

(6) $$l_{U2C}\left(z^{i}\right)=-log\frac{exp(cos\theta_{ii})}{\frac{1}{B}\sum_{j=1}^{B}exp(cos\theta_{ij})}$$

In this way, by minimizing the U2C loss, the training encourages the encoded vectors of samples with the same prototype to be as similar as possible to the prototype. This allows each component of the learned latent distribution to be responsible for a certain cluster of the data.

#### 3.3.3. Optimization Algorithm of Latent Distribution Parameters

In order to train the parameters of Gaussian mixture distribution of latent space, it is crucial to obtain the gradient of the parameters during the training. Gurumurthy et al. [16] and Ben-Yosef et al. [15] adopted the “reparameterization trick” proposed by Kingma et al. [29] in their related work to update the mean vectors $\mu_{k}$ and covariance matrices $\Sigma_{k}$ of each Gaussian component. However, the above method assumes uniform mixing coefficients $p\left(k\right)$ that are fixed. As a consequence, it fails to generate data in the case of imbalanced datasets. Based on the generalized Stein lemma, Hwang et al. [19] derived gradient identities of the parameters of Gaussian mixture distribution, which not only enables $\mu_{k}$ and $\Sigma_{k}$ to be updated, but also ensures that the mixing coefficients $p\left(k\right)$ can be updated. It is called the Stein latent optimization algorithm. The W-SLOGAN employs the Stein latent optimization algorithm to enable the imbalanced attributes to be naturally clustered in a continuous latent space. Table 4 presents a comparison of these two reparameterization techniques.

#### 3.3.4. Training Process

Step 1. Parameter initialization. Initialize parameters of the Gaussian mixture distribution, including $\mu_{k}$, $\Sigma_{k}$, and $p\left(k\right)$, as well as the parameters of three networks *G*, *D*, and *E*.

Step 2. Train D for b_D_ times. Train *D* with the discriminator objective function as presented in Equation (2).

Step 3. Train *G*, *E*, $\mu_{k}$, $\Sigma_{k},$ and $p\left(k\right)$ for one time. Train them with the total objective function as presented in Equation (4). Go to Step 2.

The loop of step 2 and step 3 stops after it is carried out for a predefined number of times.

### 3.4. Compositive Similarity Metric

**Figure 5:**
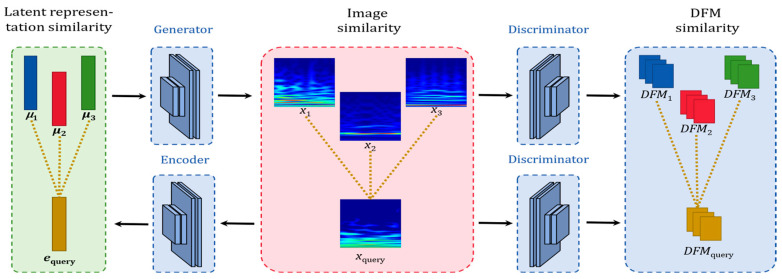
Three levels of similarity for clustering. Assume the number of Gaussian components is 3. DFM: deep feature map. $\mu_{1}$, $\mu_{2},$ and $\mu_{3}$ denote the mean vectors of the three Gaussian components, corresponding to the latent prototypes of the three clusters. $e_{query}$ denotes the latent representation of the query signal. $x_{1}$, $x_{2}$, and $x_{3}$ denote the baseline scalograms of the three clusters. $x_{query}$ denotes the scalogram of the query signal. ${DFM}_{1}$, ${DFM}_{2}$, and ${DFM}_{3}$ denote the baseline deep feature maps of the three clusters. ${DFM}_{query}$ denotes the deep feature map of the query signal.

In our clustering approach, the similarity metric plays the role of critic function, which will enable us to assign a cluster for the query data. Having the prototype as a baseline of each cluster, we put forward a compositive similarity metric that combines similarities between the test data and the prototype of a given cluster on three levels, namely latent representation, image, and deep feature map. Figure 5 illustrates three levels of similarity for clustering.

Latent representation similarity. The scalogram $x_{query}$ of the query signal is mapped to the latent space by the encoder (*E*) to become an encoded vector, which can be viewed as the latent representation of the query signal, denoted by $e_{query}=E(x_{query})$. Latent representation similarity measures similarity between $e_{query}$ and the latent prototype that is responsible for a given cluster. It is defined in (7), using cosine similarity to measure the similarity between two vectors.

(7) $$S_{latent}^{k}=\frac{e_{query}\cdot \mu_{k}}{\left\|e_{query}\right\|\left\|\mu_{k}\right\|}$$

where $S_{latent}^{k}$ denotes the similarity between the query signal and the *k*-th cluster in latent space.

Image similarity. It measures similarity between the scalogram of the query signal $x_{query}$ and the baseline scalogram $x_{k}$ of a given cluster that is generated by the generator *G* from the prototype $\mu_{k}$ of the given cluster, and is formulated by $x_{k}=G(\mu_{k})$. Image similarity is defined as follows:

(8) $$S_{image}^{k}=\frac{1}{{\left\|x_{query}-G(\mu_{k})\right\|}_{1}}$$

DFM. similarity. It measures the similarity between the DFM of the query signal, ${DFM}_{query}$, and the DFM of the baseline DFM of a given cluster, ${DFM}_{k}=DFM\left(x_{k}\right)$. DFM refers to the output of the last convolution layer of the discriminator that can be viewed as deep feature map of a given image. This kind of deep feature is inspired by the work of NHAN et al. [30], where the discriminator is employed as an unsupervised feature extractor. DFM similarity is defined as

(9) $$S_{DFM}^{k}=\frac{1}{{\left\|DFM(x_{query})-DFM\left(x_{k}\right)\right\|}_{1}}$$

Compositive similarity. We define a compositive similarity between the query signal and the centroid of the *k*th cluster that incorporates all the three levels of similarities as

(10) $$S_{com}^{k}=\alpha_{1}*S_{latent}^{k}+\alpha_{2}*S_{image}^{k}+\alpha_{3}*S_{DFM}^{k}$$

(11) $$\alpha_{1}+\alpha_{2}+\alpha_{3}=1$$

where $\alpha_{1}$, $\alpha_{2}$, and $\alpha_{3}$ are weight coefficients of the three similarities. As the dimensions are different, it is necessary to normalize $S_{latent}^{k}$, $S_{image}^{k}$, and $S_{DFM}^{k}$ separately before calculating the compositive similarity.

Through some sensitivity tests, we found that the proposed clustering approach is somehow sensitive to $\alpha_{1}\;$and $\alpha_{2}$ values. Therefore, to seek good settings for them, we carried out multiple experiments where $\alpha_{1}\;$and $\alpha_{2}$ were set to different values. Then, based on the resulting clustering performance evaluated with external clustering indexes, we determined the optimal values. In real applications where the data are unlabeled, optimal values can also be determined by use of internal clustering indexes.

The query data can then be assigned to the cluster with the highest compositive similarity (i.e., $\underset{k}{\mathrm{argmax}}S_{com}^{k}$).

With respect to computational complexity, when applied to a dataset with size *n*, the complexity of both the training and clustering of the W-SLOGAN algorithm are O(*n*), which is linear.

### 3.5. External Clustering Indexes

To evaluate the closeness between the clustering results from the proposed approach and classification of the data, three widely used external clustering indexes were adopted, namely Purity, Adjusted Rand Index (ARI), and Normalized Mutual Information (NMI).

Purity. Purity is an intuitive evaluation index that indicates the degree of agreement between the clustering results and the real data distribution. It is defined as

(12) $$Purity=\frac{1}{n}\sum_{i=1}^{k}{max}_{j}(n_{ij})$$

where *n* denotes the total number of samples. *k* denotes the total number of clusters. $n_{ij}$ denotes the number of samples in both class $u_{j}$ and cluster $v_{i}$. The range of the Purity value is [0, 1], where a higher value indicates a purer clustering result.

ARI. ARI measures the degree of similarity between two data distributions [31]. It takes into consideration the consistency between the resulted cluster labels and class labels. The definition of ARI is

(13) ARI=∑i∑jnij2−[∑iai2∑jbj2]/n212∑iai2+∑jbj2−[∑iai2∑jbj2]/n2

where $n_{ij}$ denotes the number of samples in both class $u_{j}$ and cluster $v_{i}$.$\;a_{i}$ denotes the number of samples in class $u_{i}$ and $b_{j}$ denotes the number of samples in cluster $v_{j}$. *n* is the total number of samples. nij2 represents a combination and is equal to $C_{n_{ij}}^{2}$.

NMI. NMI evaluates the consistency between two distributions by measuring their mutual information [32]. The definition of NMI is presented as follows:

(14) $$NMI(C,K)=\frac{MI(C,K)}{\sqrt{H(C)\cdot H(K)}}$$

where MI(C,K) denotes the mutual information between class labels and the resulted cluster labels, *H*(*C*) is the entropy of classification labels, and *H*(*K*) is the entropy of clustering results. The range of the NMI value is [0, 1], where 1 indicates perfect consistency, which means the clustering is exactly consistent with the classification.

### 3.6. Experimental Setup and Running Environment

The Bonn dataset consists of five subsets (A, B, C, D, and E), which were organized into four groups in the experiments. Such grouping considers the complexity of signals of each subset and the relevance of the subsets in clinical. We segmented the scalp EEG and iEEG signals from the Bonn dataset using a sliding time window of 2.88 s with 50% overlap. For each subset, all the 100 signals each with 23.6 s duration were segmented to 1500 samples each with 2.88 s duration. In the experiments, the cluster number was set according to the class number in each group. Detailed descriptions of the four groups are shown in Table 5.

The HUP dataset comprises three classes of signals: pre-ictal, ictal, and inter-ictal. The pre-ictal one is defined as two minutes before the seizure onset. The ECoG signals were segmented with a sliding time window of 5 s with 50% overlap. The number of clusters was set to 3. The description of the ECoG data of three de-identified epileptics in the HUP dataset is outlined in Table 6.

In each experimental group, all the data were used for the unsupervised training of W-SLOGAN; then, with the trained model, all the EEG segments were clustered; at last, the clustering results were evaluated with three external clustering indexes.

Preprocessing. Each signal was filtered by an FIR bandpass filter, preserving information within the frequency range from 0.5 to 40 Hz. The scalp EEGs and iEEGs from the Bonn dataset were segmented into 2.88-s segments, while the ECoG signals from the HUP dataset were segmented into 5-s segments. The 1-d time series segments were transformed to 2-d scalograms. The Morlet wavelet was selected as the mother wavelet in the continuous wavelet transform. The scalogram dimension is 64 × 64 × 3 (3 is the number of RGB channels). Before feeding these scalograms into the W-SLOGAN model for training, each pixel value of the scalograms was scaled to ensure their range fell within (−1, 1).

Parameter settings. For simplicity, we denote the learning rate of generator as η, the learning rate of the covariance $\Sigma_{k}$ as γ, the gradient penalty coefficient as $\lambda_{1}$, the weight coefficient of the U2C loss as $\lambda_{2}$, and the weights for the latent representation, image, and DFM similarities as $\alpha_{1}$, $\alpha_{2}$, and $\alpha_{3}$, respectively. In the experiments, the learning rate of discriminator was set to 4η, and the learning rate of encoder to η. The learning rate of latent prototype $\mu_{k}$ was set to 10γ, and the learning rate of mixing coefficient $p\left(k\right)$ to γ. Specifically, the parameter values were η=0.0001, γ=0.004, and λ=10. In addition, we initialized the $p\left(k\right)=1/N$, $\Sigma_{k}=I_{d_{z}\times d_{z}}$, and $\mu_{k}$ sampling from $N(0,I_{d_{z}\times d_{z}})$. The three weights $\alpha_{1}$, $\alpha_{2}$, and $\alpha_{3}$ were empirically set to 1/3.

During the training, the Adam optimizer was employed to train *G*, *D*, and *E*, and the stochastic gradient descent (SGD) optimizer was adopted to train $\Sigma_{k}$, $\mu_{k}$, and $p\left(k\right)$. The batch size (*B*) was 64 and the number of training iterations was set to 18,000 to ensure sufficient training. In the clustering experiments, we repeated each experiment several times and reported the means and standard deviations of model performances. Table 7 shows the details of the experimental parameter settings.

Running environment. The experimental conditions include a desktop computer equipped with an Inter(R) Core (TM) i9-10900K CPU (Inter, Santa Clara, CA, USA) and an NVIDIA GeForce RTX 3080 GPU (Nvidia, Santa Clara, CA, USA). Segmentation and continuous wavelet transform of the signals were implemented with MATLAB (R2019a), while the training and evaluation of W-SLOGAN were carried out with Python 3.7 and TensorFlow 2.6.0.

## 4. Results

### 4.1. Clustering Results

**Figure 6:**
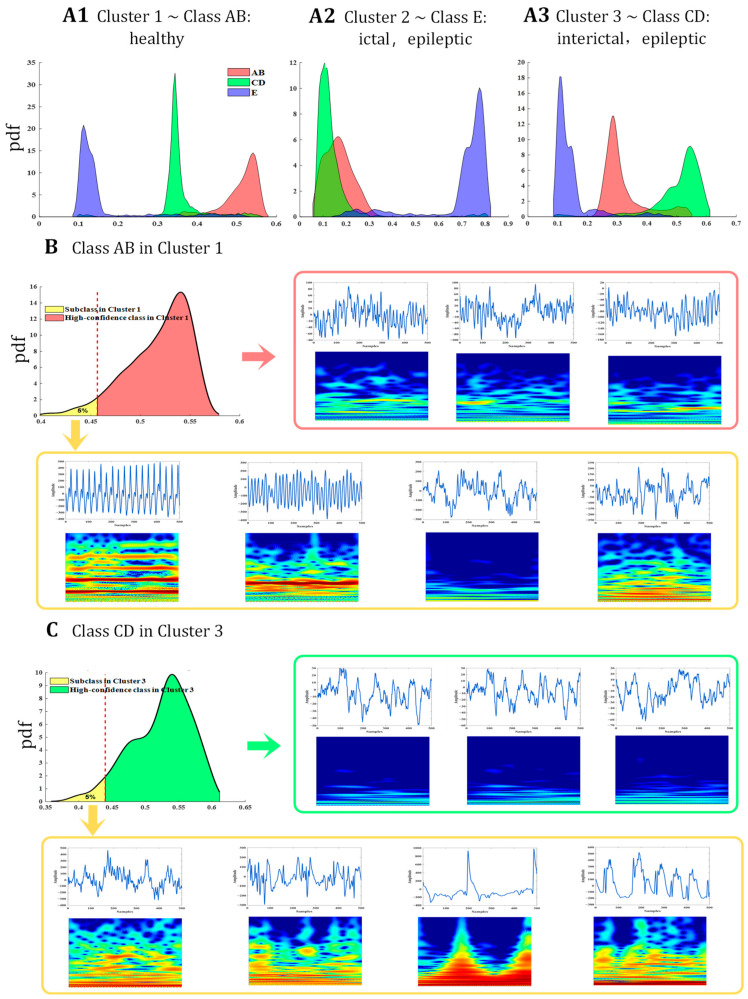
Clustering results and intra-class diversity. (**A1**–**A3**) show the probability density functions for samples belonging to Cluster 1, Cluster 2, and Cluster 3, respectively. (**B**) shows the probability density function of Class AB samples clustered into Cluster 1, several high-probability samples with their scalograms (in the upper row), and several low-probability samples with their respective scalograms (in the lower row). (**C**) shows the probability density functions of Class CD samples clustered into Cluster 3, several high-probability samples with their scalograms, and several low-probability samples with their scalograms (in the lower row).

From applying the proposed approach to the benchmark Bonn EEG datasets, the clustering results and the classification of the data are highly consistent. Taking the group AB_CD_E of the Bonn dataset as an example, the three subplots of Figure 6A depict the probability density functions for signals to belong to Cluster 1, Cluster 2, and Cluster 3. Red, green, and purple indicate samples from Class AB (healthy), Class CD (inter-ictal, epileptic) and Class E (ictal, epileptic), respectively. Taking Cluster 1 as an example, as shown in Figure 6A1, the probability that Class AB samples belong to this cluster is the highest, while the probability that Class CD and Class E samples belong to that cluster is relatively low. It indicates that Cluster 1 gathers the general samples of Class AB. In other words, Cluster 1 corresponds to class AB. Likewise, from Figure 6A2,A3, it is obvious that Cluster 2 and Cluster 3 correspond to Class E and Class CD, respectively. On the other hand, there are also some samples whose resulted clustering labels are inconsistent with their class labels. For example, a few samples of Class CD and E are clustered into Cluster 1, although the probability that they belong to that cluster is not high. It does not necessarily indicate clustering error, but rather reveals the intra-class diversity.

The samples whose resulted cluster label is consistent with its class label represent the generic attributes of that class. The following analysis will focus on this part of samples. Figure 6B shows the probability density function of Class AB samples that are clustered into Cluster 1. These samples can be divided into two parts; one comprises 95% of samples with higher probabilities, and the other comprises the other 5% with lower probabilities. The red color indicates high-probability samples. Several high-probability samples with their respective scalograms are shown in the upper row, representing the typical attributes of Class AB. The yellow color indicate low-probability samples that are shown in the lower row. Similarly, Figure 6C shows the probability density functions of Class CD samples clustered into Cluster 3, several high-probability samples, and low-probability samples. As for Class E samples, see Section 4.4 for a more detailed analysis.

It is obvious that (i) the high-probability samples of each cluster are quite similar, reflecting the typical characteristic of that cluster or class. For example, the high-probability samples of Cluster 1 reflect the characteristics of the EEG signals of healthy volunteers with eyes opened or closed, i.e., the amplitude fluctuates between −180 and 100, fluctuations with a relatively high frequency that suggest rapid changes in the brain activity. The high-probability samples of Cluster 3 reflect the characteristics of iEEG signals during inter-ictal periods of epileptics, i.e., the amplitude fluctuates between −70 and 70 with a relatively low frequency. (ii) Low-probability samples exhibit significant differences in terms of waveforms and scalograms compared to high-probability samples. They show diverse patterns. It could reflect intra-class diversity or may be caused by noise. 

### 4.2. Clustering Results from Different Similarity Metrics

According to the class labels provided in the dataset, the closeness of the resulted clustering to the data classification can be measured by three external clustering evaluation indexes, namely Purity, ARI, and NMI. In order to observe the role that different similarity plays in clustering in the four groups of EEG data of the Bonn dataset and the three epileptic patient’s ECoG data of the HUP dataset, we applied clustering separately with three kinds of similarity metrics (namely single latent representation similarity, latent representation similarity + image similarity, and the compositive similarity that incorporates all the three levels of similarities). The results are shown in Table 8 and Table 9.

Table 7 and Table 8 show that, in either the Bonn dataset or the HUP dataset, clustering by the use of the compositive similarity outperforms that of the other two kinds of similarities. Specifically, clustering using the compositive similarity achieves the best average rank (1.43) when evaluated with Purity, ARI, and NMI. Also, the compositive similarity achieved the largest number of the best Purity, ARI, and NMI out of the seven groups of experiments (five out of seven).

**Figure 7:**
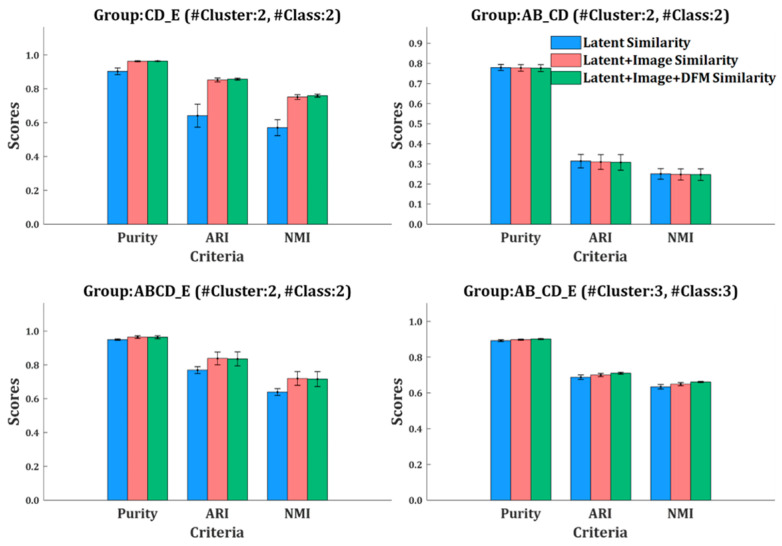
Purity, ARI, and NMI of the results of clustering on four groups of EEG/intracranial EEG (iEEG) data of the Bonn dataset separately using different kinds of similarities.

**Figure 8:**
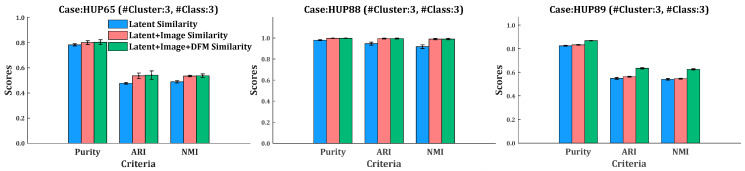
Purity, ARI, and NMI of the results of clustering on three epileptic subjects of ECoG data of the HUP dataset separately using different kinds of similarities.

Figure 7 and Figure 8 show the bar charts of the three external clustering indexes for clustering using different kinds of similarities on the Bonn dataset and the HUP dataset, respectively. Compared to using a single latent representation similarity, the inclusion of image similarity significantly improved the clustering performance in most experimental groups. In the Bonn dataset, the average Purity, ARI, and NMI increased by 2.18%, 11.92%, and 13.04%, respectively. In the HUP dataset, those three indexes averagely increased by 1.70%, 6.20%, and 6.36%, respectively. The improvement in the performance implies that the scalogram has the potential to capture the time-frequency characteristics of different EEG signals. However, compared to the combination of latent representation similarity and image similarity, the inclusion of DFM similarity has little effect on the improvement in clustering performance. In the Bonn dataset, the three indexes increased by 0.1%, 0.37%, and 0.66%, respectively.

### 4.3. W-SLOGAN’s Training

#### 4.3.1. Impact of the Number of Iterations in Training W-SLOGAN

**Figure 9:**
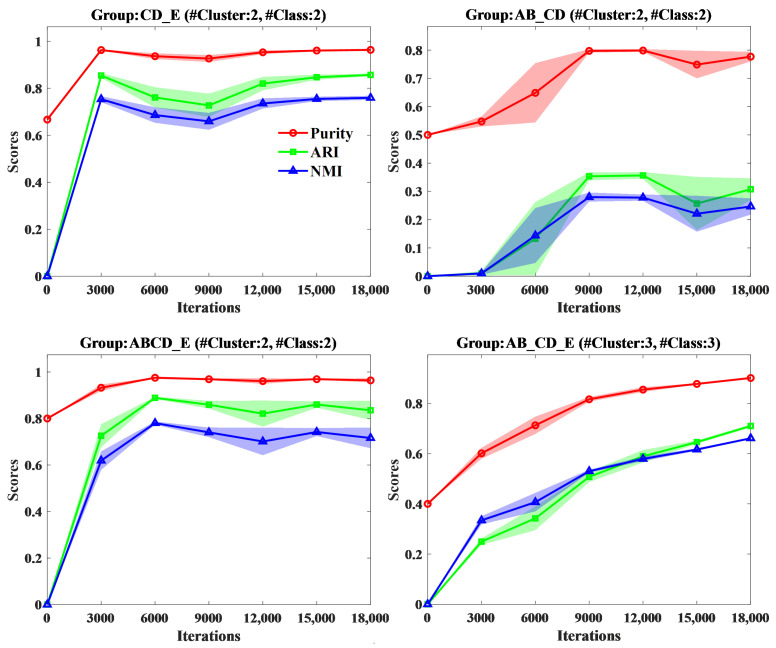
Impact of the iteration number during training W-SLOGAN on the clustering performance on four groups of EEG data of the Bonn dataset separately evaluated with Purity, ARI, and NMI.

**Figure 10:**
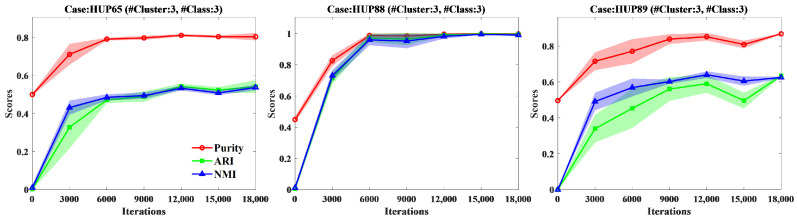
Impact of the iteration number duringtraining W-SLOGAN on the clustering performance on three epileptic subjects of ECoG data of the HUP dataset separately evaluated with Purity, ARI and NMI.

We investigated the impact of the number of iterations during the training on the clustering performance of the W-SLOGAN model. We evaluated the clustering performance of the proposed approach on the two datasets separately when the model was iterated 0, 3000, 6000, 9000, 12000, 15,000, and 18,000 times, respectively. The results are shown in Figure 9 and Figure 10. It is shown that, in most groups, a fairly good clustering performance was achieved by 9000 iterations. Before that, the performance increases rapidly with the increase in the iteration number, whereas after that, it changes smoothly. Nevertheless, for part of groups such as AB_CD_E, the clustering performance continues to increase with the increase in the iteration number. 

#### 4.3.2. Reproducibility of the Results

The results are to some extent reproducible. It is based on our experiments to test the reproducibility on each experimental group. Taking the group of AB_CD_E of Bonn dataset as example, we trained three W-SLOGAN models with the same experimental setup and parameters. The mixing coefficients *p*(*k*) of the latent mixture components obtained from different trained models are displayed in Table 10. It can be seen that they are close. Also, the ratios of the Gaussian mixture components are all close to 2:2:1. They fit the true class ratio (3000:3000:1500) of that group.

### 4.4. Exploratory EEG Analysis

#### 4.4.1. Discovery of Different Types of Epileptiform Waves

In order to explore the diversity of ictal iEEG signals, we carried out clustering on the ictal data of the Bonn dataset. The number of clusters was set to five. It is noteworthy that the three resulting clusters were highly consistent with three typical kinds of epileptiform waves in the characteristic patterns. Epileptic seizures are accompanied by some typical discharge waveforms, which serve as significant characteristics and diagnostic criteria for epileptic seizures. Common epileptiform waves include sharp wave, spike wave, spike and slow wave complex, sharp and slow wave complex, highly rhythmic disorganization, and so on. We found several clusters that correspond to rhythmic sharp wave, spike and slow wave complex, and highly rhythmic disorganization from the iEEG recordings.

**Figure 11:**
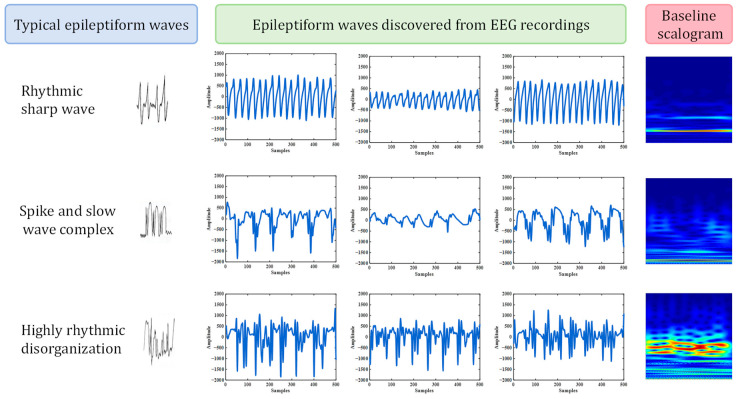
Typical kinds of epileptiform waveforms were found by clustering the ictal iEEG data of the Bonn dataset. In each row are displayed the characteristic waveform of a type of epileptiform discharge, three epileptiform waves of that type that were clustered into a same cluster found from the iEEG recordings by our approach, as well as the baseline scalogram of that cluster.

Figure 11 shows the three types of epileptiform waveforms and the corresponding clusters that resulted from our approach. On each row are displayed the characteristic waveform of a type of epileptiform discharge, three epileptiform waves of that type that were clustered into a same cluster found from the iEEG recordings by our approach, as well as the baseline scalogram of that cluster.

For reference, the definitions and characteristics of sharp wave, spike and slow wave complex, and highly rhythmic disorganization are listed below [33].

Sharp wave. Sharp waves are the most basic form of burst EEG activity, lasting from 70 to 200 ms (5–14 Hz). The amplitudes range from 100 to 200 μV. They are usually with the form of negative phase waves.

Spike and slow wave complex. A pattern of epileptiform waveform composed of spike waves and slow waves. The slow wave is the predominant component of this complex, lasting approximately from 200 to 500 ms. The spike and slow wave complex typically has higher amplitudes, ranging from 105 to 300 μV, and can reach even more than 500 μV.

Highly rhythmic disorganization. Usually composed of sharp waves, spikes, etc., the frequency and amplitude are highly irregular, often seen in complex partial seizures.

#### 4.4.2. Multiple Labels of EEG Data

**Figure 12:**
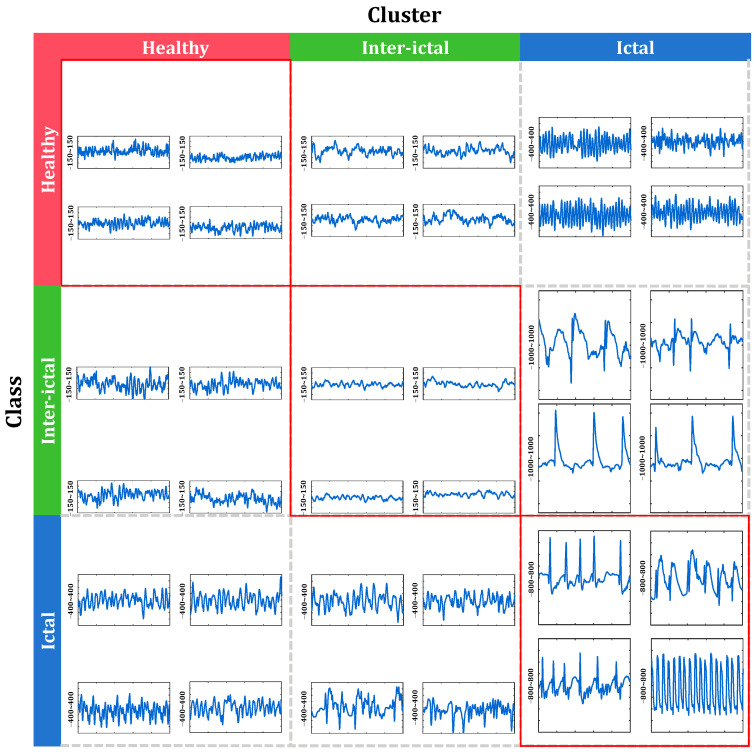
Class labels and clustering results of several samples in group AB_CD_E of the Bonn dataset. Samples on each row belong to a same class and those on each column are clustered into a same cluster. Each grid displays four samples. Row 1 and column 1 both correspond to Class AB, i.e., healthy; Row 2 and column 2 both correspond to Class CD, i.e., inter-ictal, epileptic; Row 3 and column 3 both correspond to Class E, i.e., ictal, epileptic.

The cross-analysis of clustering and classification has the potential to discover interesting knowledge, including multiple labels of data. Taking the group AB_CD_E of the Bonn dataset as an example, Figure 12 shows the class labels and clustering results of several samples. Samples in each row belong to a same class and those in each column are clustered into a same cluster. Each grid displays four samples. Row 1 and column 1 both correspond to Class AB, i.e., healthy; Row 2 and column 2 both correspond to Class CD, i.e., inter-ictal; and Row 3 and column 3 both correspond to Class E, i.e., inter-ictal.

The class labels of waveforms on the diagonal in the Figure 12 are consistent with their respective cluster labels. These waveforms best reflect the salient attributes of that type of EEG signal. For example, the samples in row 1, column 1 (AB) represent scalp EEG in healthy volunteers, showing high frequency components compared to the inter-ictal iEEG (CD) in row 2, column 2. The four ictal iEEG signals in row 3, column 3 (E) exhibit various morphology, including some typical epileptiform discharge waveforms such as rhythmic sharp waves and spike and slow wave complexes. 

Perhaps, it is signals whose class label and cluster label do not refer to the same attribute that deserve more attention. Taking the waveforms in row 2 and column 3 as an example, they belong to the inter-ictal, epileptic class; however, they are clustered as an ictal, epileptic cluster. They exhibit characteristics of typical epileptiform waveforms, such as spike waves and spike and slow wave complex. These findings are consistent with the clinical experience about the existence of epileptiform discharges in inter-ictal periods. In fact, these signals with multiple attributes should be annotated with multiple labels so that the information within the recordings can be reflected more comprehensively and objectively.

## 5. Discussion

Clustering plays a unique role in exploratory EEG analysis. It is unsupervised, and consequently, has low labor and time costs. With our approach, the adaptively learned Gaussian mixing coefficients make the model remain effective in dealing with imbalanced datasets. By means of intra-class clustering or cross-analysis of clustering and classification, it is possible to reveal intra-class diversity or other interesting information. As demonstrated in this work, the proposed approach is attractive for practice in epileptic subtype diagnosis, multiple labelling of EEG data, etc.

With the latent prototype-based clustering approach, the clustering results are close to the classification of the data (with reference to the results in Section 4.1 and Section 4.2). It is in part due to the sound definition of the critic function, which is based on latent prototypes and measures the similarity on three levels. In this way, the approach is able to detect underlying unknown patterns in the data. Nevertheless, we would like to point out that, even if the clustering result is inconsistent with the classification, it does not mean that the performance of the clustering method is not good. This is because the objectives of clustering and classification are different. Classification is task-oriented, while clustering organizes data elements according to the resemblance of the intrinsic patterns (if any) of the data.

Different types of epileptiform waves were discovered from EEG recordings, as shown in Figure 11, in an unsupervised way without any given type label. It has been found that some types of epileptic waveforms are related to specific epilepsy subtypes. For example, rhythmic sharp waves are often associated with focal seizures, while the spike and slow wave complex is more common in absence seizures. Therefore, our approach can not only reveal the diversity of EEG signals during seizures and provide with a representative scalogram of each subtype, but also can point out when and what type of epileptic discharge occurs in the brain so as to assist the doctor in epilepsy subtype diagnosis.

Multiple labels of EEG or iEEG data can be discovered by means of the cross-analysis of clustering and classification, as shown in Figure 12. Such cross-analysis between unknown kinds and known classes has the potential to reveal novel knowledge. Sometimes, it is the signal whose class label and cluster label do not refer to the same attribute that deserve more attention. They reflect intra-class diversity. On the other hand, such analysis helps to better understand multiple attributes of the data. As revealed in Figure 12, in ictal-period iEEG signals of an epileptic, there exist waveforms similar to that of a healthy subject, while in the interictal period, some epileptiform discharges are found. In fact, these signals with multiple attributes should be annotated with multiple labels so that the information within the recordings can be reflected more comprehensively and objectively. 

Discussion on DFM similarity. As shown in Figure 7 and Figure 8, the inclusion of DFM similarity has little effect on the improvement in clustering performance. This may be due to the fact that the discriminator’s task is to distinguish between real and fake samples. The features extracted by its convolutional layer are those that focus on that task. Hence, in most cases, the DFM level similarity plays a less important role in clustering than the other two levels of similarity. But, in a few cases, e.g., ECoG of HUP89, the use of DFM similarity is more effective than that of image similarity. This detail problem is pending for further study.

With respect to the W-SLOGAN model, the number of Gaussian components of the latent distribution need to be set in advance according to a predetermined number of clusters. The optimization of the cluster number may be a future research direction.

## Figures and Tables

**Table 1:** Description of the Bonn dataset.

Subjects	Set A	Set B	Set C		Set D	Set E
	Healthy Volunteers			Epileptic Patients		
Patient state	Eyes open	Eyes closed	Inter-ictal		Inter-ictal	Ictal
Electrode types	Surface	Surface	Intracranial		Intracranial	Intracranial
Electrode placement	International 10/20 systems	International 10/20 systems	Opposite epileptogenic zone		Within epileptogenic zone	Within epileptogenic zone
No. of samples	100	100	100		100	100
Sampling points	4096	4096	4096		4096	4096

**Table 2:** Description of electrocorticography (ECoG) data of three patients in the HUP dataset.

Patients	Gender	Age	Target	Therapy	Electrode
HUP65	M	36	Temporal	Resection	RG 11-Ref
HUP88	F	35	Temporal	Resection	LMST 02-Ref
HUP89	M	29	Temporal	Resection	AD 04-Ref

**Table 3:** Implementation details of the W-SLOGAN model.

Network	Layer (Type)	Maps	Size	Kernel Size	Activation	BN ^a^ Layer
Generator	Input_1	None	100	None	None	None
	Dense	None	8192	None	None	None
	Reshape	512	4 × 4	None	ReLU	yes
	ConvTranspose2D	256	8 × 8	5 × 5	ReLU	yes
	ConvTranspose2D	128	16 × 16	5 × 5	ReLU	yes
	ConvTranspose2D	64	32 × 32	5 × 5	ReLU	yes
	ConvTranspose2D	3	64 × 64	5 × 5	ReLU	yes
Discriminator	Input_2	3	64 × 64	None	None	None
	Conv2D	64	32 × 32	5 × 5	LeakyReLU	None
	Conv2D	128	16 × 16	5 × 5	LeakyReLU	None
	Conv2D	256	8 × 8	5 × 5	LeakyReLU	None
	Conv2D	512	4 × 4	5 × 5	LeakyReLU	None
	Flatten	None	8192	None	None	None
	Dense	None	1	None	None	None
Encoder	Input_3	3	64 × 64	None	None	None
	Conv2D	64	32 × 32	5 × 5	ReLU	yes
	Conv2D	128	16 × 16	5 × 5	ReLU	yes
	Conv2D	256	8 × 8	5 × 5	ReLU	yes
	Conv2D	512	4 × 4	5 × 5	ReLU	yes
	GAP ^b^	None	512	None	None	None
	Dense	None	100	None	None	None

^a^ Batch Normalization. ^b^ Global Average Pooling.

**Table 4:** Comparison of two reparameterization methods.

Models	Reparameterization Form	Trainable Parameters	Characteristics of Gradient Estimation
AEVB [29] DeLiGAN [16] GM-GAN [15]	Explicit	$\mu_{k}$ $\;\mathrm{and}\;\Sigma_{k}$	Unbiased; high variance
SLOGAN [19]	Implicit	$\mu_{k}$ $,\;\Sigma_{k}$ and p(k)	Unbiased; low variance

**Table 5:** Description of four experimental groups of the Bonn dataset.

Group	Set	Description	# Class	# Cluster	Class Ratio
CD_E	Sets C and D versus Set E	Inter-ictal and ictal	2	2	3000:1500
AB_CD	Sets A and B versus Sets C and D	Healthy and inter-ictal	2	2	3000:3000
ABCD_E	Sets A, B, C, and D versus Set E	Non-seizure and seizure	2	2	6000:1500
AB_CD_E	Sets A and B versus Sets C and D versus Set E	Healthy, inter-ictal, and ictal	3	3	3000:3000:1500

**Table 6:** Description of the ECoG data of three de-identified epileptics in the HUP dataset.

Case	Description	# Class	# Cluster	# Pre-Ictal	# Ictal	# Inter-Ictal
HUP65	pre-ictal, inter-ictal, and ictal	3	3	348	592	251
HUP88	pre-ictal, inter-ictal, and ictal	3	3	348	592	724
HUP89	pre-ictal, inter-ictal, and ictal	3	3	348	592	252

**Table 7:** Details of the experimental parameter settings.

Parameters	Initialization	Optimizer	Learning Rate
Generator	Random	Adam	0.0001
Discriminator	Random	Adam	0.0004
Encoder	Random	Adam	0.0001
$\mu_{k}$	$N(0,I_{d_{z}\times d_{z}})$	SGD	0.04
$\Sigma_{k}$	$I_{d_{z}\times d_{z}}$	SGD	0.004
$p\left(k\right)$	1/N	SGD	0.004
$\lambda_{1}$	10	None	None
$\lambda_{2}$	1	None	None
Batch size	64		
Iterations	18,000		

**Table 8:** The clustering results of the Bonn dataset by the use of different kinds of similarity metrics.

Group	Criteria	Latent Representation Similarity	Latent Representation + Image Similarity	Latent Representation + Image + DFM Similarity
CD_E # Cluster:2 # Class:2	Purity	0.9033 ± 0.0200	0.9620 ± 0.0030	0.9633 ± 0.0015
	ARI	0.6410 ± 0.0680	0.8518 ± 0.0115	0.8568 ± 0.0056
	NMI	0.5704 ± 0.0472	0.7510 ± 0.0137	0.7592 ± 0.0089
AB_CD # Cluster:2 # Class:2	Purity	0.7798 ± 0.0147	0.7778 ± 0.0161	0.7768 ± 0.0172
	ARI	0.3139 ± 0.0335	0.3096 ± 0.0365	0.3076 ± 0.0389
	NMI	0.2503 ± 0.0265	0.2475 ± 0.0277	0.2466 ± 0.0288
ABCD_E # Cluster:2 # Class:2	Purity	0.9494 ± 0.0043	0.9644 ± 0.0081	0.9638 ± 0.0089
	ARI	0.7694 ± 0.0199	0.8382 ± 0.0377	0.8354 ± 0.0412
	NMI	0.6396 ± 0.0199	0.7199 ± 0.0408	0.7162 ± 0.0442
AB_CD_E # Cluster:3 # Class:3	Purity	0.8925 ± 0.0048	0.8977 ± 0.0032	0.9015 ± 0.0020
	ARI	0.6882 ± 0.0124	0.7003 ± 0.0088	0.7102 ± 0.0055
	NMI	0.6341 ± 0.0125	0.6491 ± 0.0090	0.6613 ± 0.0031
Avg Purity Avg Purity Rank # Best Purity		0.8813 2.5 1	0.9005 1.75 1	0.9014 1.75 2
Avg ARI Avg ARI Rank # Best ARI		0.6031 2.5 1	0.6750 1.75 1	0.6775 1.75 2
Avg NMI Avg NMI Rank # Best NMI		0.5236 2.5 1	0.5919 1.75 1	0.5958 1.75 2

# Best Purity, # Best ARI, and # NMI, respectively, indicate the largest number of the best Purity, ARI, and NMI across four groups of the Bonn dataset.

**Table 9:** The clustering results of the HUP dataset by the use of different kinds of similarity metrics.

Case	Criteria	Latent Representation Similarity	Latent Representation + Image Similarity	Latent Representation + Image + DFM Similarity
HUP65 # Cluster:3 # Class:3	Purity	0.7834 ± 0.0086	0.8013 ± 0.0152	0.8044 ± 0.0195
	ARI	0.4774 ± 0.0067	0.5368 ± 0.0222	0.5421 ± 0.0336
	NMI	0.4893 ± 0.0096	0.5356 ± 0.0064	0.5375 ± 0.0148
HUP88 # Cluster:3 # Class:3	Purity	0.9804 ± 0.0042	0.9982 ± 0.0017	0.9982 ± 0.0015
	ARI	0.9471 ± 0.0144	0.9956 ± 0.0041	0.9956 ± 0.0036
	NMI	0.9180 ± 0.0175	0.9904 ± 0.0078	0.9905 ± 0.0074
HUP89 # Cluster:3 # Class:3	Purity	0.8249 ± 0.0039	0.8333 ± 0.0024	0.8686 ± 0.0020
	ARI	0.5483 ± 0.0076	0.5627 ± 0.0046	0.6344 ± 0.0054
	NMI	0.5408 ± 0.0069	0.5460 ± 0.0036	0.6253 ± 0.0052
Avg Purity Avg Purity Rank # Best Purity		0.8629 3.0 0	0.8776 1.6667 1	0.8904 1.0 3
Avg ARI Avg ARI Rank # Best ARI		0.6576 3.0 0	0.6984 1.6667 1	0.7240 1.0 3
Avg NMI Avg NMI Rank # Best NMI		0.6494 3.0 0	0.6907 2.0 0	0.7178 1.0 3

# Best Purity, # Best ARI, and # NMI, respectively, indicate the largest number of the best Purity, ARI, and NMI across three epileptic subjects of the HUP dataset.

**Table 10:** Mixing coefficients *p*(*k*) of the latent mixture components obtained from different trained W-SLOGAN models.

Model	Model_1	Model_2	Model_3
$p\left(k=1\right)$	0.3869	0.3969	0.373
$p\left(k=2\right)$	0.448	0.439	0.4627
$p\left(k=3\right)$	0.1651	0.1641	0.1643

## Data Availability

The original data presented in the study are openly available in the Bonn Dataset at https://www.ukbonn.de/epileptologie/arbeitsgruppen/ag-lehnertz-neurophysik/downloads/ (accessed on 12 April 2024) and in the HUP iEEG Epilepsy Dataset at https://openneuro.org/datasets/ds004100/versions/1.1.3 (accessed on 2 May 2024).
